# Improvement of Antioxidant Activity and Sensory Properties of Functional Cookies by Fortification with Ultrasound-Assisted Hot-Air-Drying Blackberry Powders

**DOI:** 10.3390/foods13152402

**Published:** 2024-07-29

**Authors:** Pasquale Roppolo, Carla Buzzanca, Angela D’Amico, Alessandra Culmone, Ilenia Tinebra, Roberta Passafiume, Sonia Bonacci, Vittorio Farina, Vita Di Stefano

**Affiliations:** 1 Department of Agricultural, Food and Forest Sciences (SAAF), University of Palermo, 90133 Palermo, Italy; pasquale.roppolo@unipa.it (P.R.); alessandra.culmone@unipa.it (A.C.); ilenia.tinebra@unipa.it (I.T.); roberta.passafiume@unipa.it (R.P.); vittorio.farina@unipa.it (V.F.); 2 Department of Biological, Chemical, and Pharmaceutical Sciences and Technologies, University of Palermo, 90133 Palermo, Italy; angela.damico02@unipa.it (A.D.); vita.distefano@unipa.it (V.D.S.); 3 Department of Health Sciences, University Magna Græcia of Catanzaro, Germaneto, 88100 Catanzaro, Italy; 4 National Biodiversity Future Center (NBFC), 90133 Palermo, Italy

**Keywords:** ultrasound-assisted hot air drying, blackberry powder, physicochemical properties, antioxidant properties, polyphenols, sensory evaluation, fortified foods

## Abstract

In response to the global challenge of food wastage and high perishability of blackberries, this study evaluated the use of ultrasound-assisted hot air drying (US-HAD) to convert downgraded blackberries into powders, comparing it with traditional hot air drying (HAD). US-HAD reduced the drying time and achieved a final moisture content of 12%. Physicochemical analyses (colourimetry, total soluble solids, titratable acidity, and total phenolic content) were conducted on fresh fruit, powders, and fortified cookies. US-HAD cookies exhibited promising antioxidant activity, with ABTS values ranging from 8.049 to 8.536 mmol TEAC/100 g and DPPH values from 8.792 to 9.232 mmol TEAC/100 g, significantly higher than control cookies. The TPC was 13.033 mgGAE/g in HAD cookies and 13.882 mgGAE/g in US-HAD cookies. UHPLC-ESI-MS analysis showed an increase in phenolic compounds content in fortified cookies compared to the control. Sensory analysis highlighted a superior blackberry flavour and overall acceptability in US-HAD cookies, with statistical analysis confirming their superior nutritional and sensory qualities. Integrating US-HAD blackberry powder into cookies helps reduce food waste and enhances the nutritional profiles of baked goods, offering functional foods with health benefits. This work provides a scientific basis for developing enriched functional cookies, offering a healthy and sustainable alternative for utilising damaged fruits.

## 1. Introduction

Over the past decade, consumer interest in foods rich in beneficial nutrients and bioactive compounds that maintain integrity and freshness has grown significantly [1]. Numerous studies [2,3,4,5] have demonstrated the health benefits of diets rich in fruits and vegetables due to the presence of phenolic compounds, which are associated with cancer prevention, cardiovascular health, and immune system enhancement.

The blackberry (*Rubus* spp.-Fam. *Rosaceae*) is of Asian origin and is cultivated in Europe, North America, and the temperate regions of Brazil [6]. This fruit is mainly found in the temperate and cool–temperate areas of the Northern Hemisphere but is also found in some warm and subtropical areas [7]. Ripe blackberries have a dark red to dark blue colour with a smooth, shiny skin [8]. They are climacteric, seasonal, and highly perishable fruits [9]. Small fruit crops, such as blackberries, are known for their antioxidant potential due to the presence of various naturally occurring active compounds. These include polyphenols, isoprenoids, and organic sulphur compounds [10,11,12]. In red berries, the most abundant compounds are phenolic acids, tannins, and flavonoids, particularly anthocyanins, which give the fruits a sour taste and attractive colour. The presence of these compounds, especially anthocyanins, makes these fruits useful in preventing diseases such as diabetes, cancer, and degenerative conditions [13]. There are quality standards for blackberries in the food industry that concern size, consistency, flavour, and nutritional content. Quality criteria include physical and chemical parameters such as a uniform colour, the absence of physical damage or contamination, and proper ripeness. The sugar content in blackberries can vary from 8 to 12 g per 100 g of fresh fruit, while the total acid content usually ranges from 0.6 to 1.5 g per 100 g, depending on the variety and growing conditions [14].

Blackberries are usually consumed fresh but can be processed and marketed frozen or as juice concentrate. In industry, blackberries that do not meet fresh market standards are used to produce food supplements, juices, yoghurt, ice cream, jellies, and other candies [15,16]. Additionally, they could be processed into semi-finished foods, such as vegetable powders, and then used in other preparations, allowing the creation of ‘fortified foods’, with bioactive compounds that offer additional health benefits [17]. This approach opens new possibilities for the use of small fruits in the food industry. For example, Pereira et al. (2019) [18] reported a study on the use of freeze-dried blackberry flour and whole blackberries for the creation of cookies while Różyło et al. (2019) [19] reported the use of freeze-dried blackberry powder in the formulation of gluten-free crispy bread. However, no study has focused on the use of blackberry powder from downgraded fruits, obtained by hot-air and ultrasound-assisted drying, as ingredients for the preparation of cookies.

Globalisation has led to overproduction and overdistribution, causing significant food waste [20]. Addressing this challenge requires the development of food technologies that preserve both the quality and quantity of the product, ensuring efficient resource utilisation. Despite their potential commercial, blackberries’ high perishability and rapid post-harvest respiration significantly compromise their nutritional and microbiological quality, limiting their shelf life and resulting in the downgrading of the fruit in world markets, leading to increased food waste. It is estimated that around 931 million tons of food are wasted globally each year, and blackberries, being highly perishable, contribute significantly to this problem. This waste not only represents a loss of valuable resources but also incurs considerable economic costs. The economic burden of food waste globally amounts to approximately USD 936 billion annually [21]. Therefore, it is crucial to develop new products using downgraded blackberry fruits to mitigate this phenomenon. 

The integration of vegetable powders into cookies represents an innovative and advantageous strategy for the food industry [22]. Cookies are a highly favoured treat, but their high fat and sugar content can pose significant health risks. With the global rise in obesity, diabetes, and cardiovascular diseases, there is increasing attention towards reducing the intake of unhealthy fats and sugars in the diet. The use of such powders allows for a significant reduction in fat and sugar content in the final products, thereby addressing the growing consumer concern for health and well-being [23]. Additionally, the use of natural ingredients with low sugar and fat content can enhance a product’s image and positively respond to market trends toward more sustainable and nutritious foods.

Among the technologies for reusing products that are not suitable for the fresh market or waste products, convective hot air drying is suitable for obtaining vegetable powders that can be used as supplements or ingredients to enrich the diet with nutrients. Drying is an ancient method that reduces water content to a final concentration that ensures microbial stability and preservation (10–14% relative humidity) [24]. Compared to other methods, hot air drying produces a qualitatively superior product using low temperatures and short treatment times, as demonstrated by Tinebra et al. (2022) in a study conducted on some Loquat varieties [25]. This technology can improve processing efficiency, preserve nutritional quality, and reduce the environmental impact of processing operations compared to other techniques such as freeze-drying, which requires much higher energy expenditure [26]. During the drying of fruits and vegetables, numerous physicochemical changes occur, such as colour alterations due to enzymatic or non-enzymatic browning reactions, texture changes, shrinkage, and the loss or degradation of nutritional compounds (e.g., ascorbic acid, carotenoids, phenolic compounds) [27]. Si et al. (2016) [28] demonstrated that the use of low-temperature freeze-drying on raspberries causes a more significant degradation of polyphenols compared to hot air drying (HAD) for 9 h. This result is understandable, considering the long drying time of 36 h required by freeze-drying. Therefore, selecting the appropriate time/temperature parameters or applying pre-treatments such as ultrasound is crucial to minimize the loss of these compounds [29]. For example, studies on the hot air drying of apples have shown that using low temperatures and short processing times can preserve vitamin C content and improve the sensory quality of the final product [30]. Kalra and Bhardwaj (2012) [31] have demonstrated that convective drying is faster and more efficient than solar drying for fruits such as mangoes, papayas, and apricots.

Some technologies can be combined with drying to facilitate water diffusion and reduce the processing time. Ultrasound pre-treatment has been shown to cause the extreme expansion and contraction of the fruit structure, making it resemble a ‘sponge’ [32]. This spongy structure accelerates the removal and evaporation of moisture compared to the control condition, thus preserving the bioactive compounds and nutritional characteristics of the fruit [33]. Several studies [34,35,36,37] have demonstrated that ultrasound improves drying efficiency by shortening processing times. When ultrasound interacts with vegetal material, it induces molecular and microscopic vibrations within [38], leading to the formation of micro-cavitations—tiny air bubbles within the matrix. These bubbles subsequently collapse, causing microscopic explosions that alter the structure and composition of the plant material [39]. A reduced drying time offers significant benefits including improved energy efficiency and a reduced environmental impact. Ultrasound pre-treatment has been applied to strawberries, significantly improving anthocyanin retention and antioxidant capacity [40]. These examples show how drying technologies and pre-treatments can be adapted to different types of fruits while maintaining their nutritional and functional properties.

The aim of this study was to evaluate the use of hot air drying assisted by ultrasound to convert damaged and non-marketable blackberries into vegetable powders for use as additives in cookie production. The study focused on assessing the impact of the drying process on the quality of the powders and their effects on the sensory characteristics of the resulting cookies.

## 2. Materials and Methods

### 2.1. Plant Material

Blackberries fruits (4 kg) not suitable for the fresh market from the farm ‘A piccoli frutti’ in Marsala (coordinates 38°6′44.859″ N, 13°20′47.063″ E), Trapani (Italy) were used for the experimentation. The fruits were transported to the post-harvest laboratory of the Agricultural Food and Forestry Sciences Department of the University of Palermo. The following parameters were measured on 50 fruits (300 g) in a single repetition for fruit, representative of the sample: weight (g), colour (RGB colour model), soluble solids content (CSS-°Brix), titratable acidity (AT-g/L^−1^ citric acid), and dry residue (%RS). These parameters were calculated to characterise the fruits before they were processed. 

### 2.2. Chemicals and Reagents

Methanol, sodium hydroxide, hydrochloric acid, sodium carbonate, sodium bicarbonate, gallic acid, Folin–Ciocalteu’s phenol reagent, and DPPH (2,2-diphenyl-1-picrylhydrazyl) were purchased from Sigma-Aldrich (Sigma-Aldrich, St. Louis, MO, USA). ABTS (2,2′ azino-bis(3-ethylbenzothiazoline-6-sulfonic-acid), potassium persulphate, and Trolox (6-hydroxy-2,5,7,8-tetramethylchroman-2-carboxylic acid) were obtained from Fluka (Buchs, Switzerland). N-hexane, ethyl acetate, potassium hydroxide, and 0.45 µm PTFE syringe filter were purchased from Thermo Fisher Scientific Inc. (Thermo Fisher Scientific, Waltham, MA, USA). Ethyl myristate, quercetin, rutin, kaempferol, naringenin, nicotinic acid, cyanidin 3,5-diglucoside, caffeic acid, ferulic acid, *p*-coumaric acid, m-coumaric acid, and chlorogenic acid were purchased from Sigma-Aldrich (St. Louis, MO, USA). ABTS (2,2′ azino-bis(3-ethylbenzothiazoline-6-sulfonic-acid), potassium persulphate, and Trolox (6-hydroxy-2,5,7,8-tetramethylchroman-2-carboxylic acid) were obtained from Fluka (Buchs, Switzerland. HPLC-grade water was obtained by purifying double distilled water in a Milli-Q Gradient A10 system (Millipore, Bedford, MA, USA).

### 2.3. Experimental Design

#### 2.3.1. Drying Design

To obtain a dried product with a residual moisture content of 12% that ensures microbiological stability [24,41,42], two different treatments were tested:

- HAD (hot air drying) 75 °C;
- US-HAD (ultrasound hot air drying) 75 °C.

Before drying, the fruits were washed and disinfected in an aqueous solution containing distilled water at 25 ± 1 °C and 200 μL/L^−1^ NaClO (5–6.5% NaClO solution, CHEMLAB) for 10 min. The samples were then divided into two lots (each 2 kg) that were homogeneous in terms of size, colour, and weight. The first lot of blackberries, after washing, was placed in a dryer as described by Roppolo et al. (2023) [43]. The second lot underwent pre-treatment in an ultrasound bath (US) (DU-32, ARGOlab, Modena, Italy) by dipping and then drying. The US treatment protocol involved heating the water to a temperature of 30 °C and then dipping the fruits in the ultrasound bath for 30 min at 22 kHz and 70W. The drying temperature was chosen based on preliminary tests and literature concerning the drying of similar fruits [28,44,45,46]. During the drying process, the weight of the trays was measured every two hours to ensure the process stopped when a residual moisture content of 12% was achieved, preventing the loss of bioactive compounds. After drying, the weight was measured to determine the dry residue (%RS) and the colour to determine the degree of browning of the fruit.

Finally, the two lots were ground in an ultra-centrifugal mill (Fritsch, Pulverisette 14, Lainate, Italy) at 10,000 rpm for 10 s to obtain vegetable powders with a suitable grain size (800 ± 50 µm), labelled as follows:

- HAD-BP (blackberry powder)
- US-HAD-BP (blackberry powder)

The powders were colour-detected and stored in hermetically sealed polyamide/polyethylene (PA/PE) bags at room temperature (20 ± 1 °C).

#### 2.3.2. Cookie Design

Vegetable powders, HAD-BP and US-HAD-BP, were used as ingredients for shortbread cookies. The preparation protocol involved adding the powders at a rate of 10% to “00”-type flour (CTRL-P) [47,48]. The flours were mixed as follows:

- HAD-BP 10% + “00” flour 90%;
- US-HAD-BP 10% + “00” flour 90%;
- CTRL-P “00” flour 100%.

The dough was prepared by adding eggs, yeast, and vegetable oil and mixing all ingredients for 3 min with a planetary mixer (model XBM10S; Electrolux Professional SpA, Pordenone, Italy) at Speed 4. The dough was stored at 4 °C for 3 h and 30 min before tempering at room temperature (20 ± 1 °C). A stainless-steel rolling pin was used to roll the dough to a target thickness (3 ± 1 mm). The dough was then cut into 30 ± 1 mm square shapes, placed on a baking tray, and baked in a laboratory oven (Compact Combi, Electrolux, Pordenone, Italia) at 165 °C and a time of 20 min. The resulting cookies were divided as follows:

- HAD-Cookies (HAD-BP 10% + “00” flour 90%):
- US-HAD-Cookies (US-HAD-BP 10% + “00” flour 90%):
- CTRL-C (“00” flour 100%).

The cookies were first left to cool at room temperature (20 ± 1 °C) for 2 h and then packed in hermetically sealed polyamide/polyethylene (PA/PE) bags under passive modified atmosphere conditions (21% O_2_ and 0.04% CO_2_) at room temperature (20 ± 1 °C) for 10 days.

### 2.4. Chemical-Physical Analysis

The weight of the fruits (g), fresh and dried, was measured with a precision electronic balance (Gibertini EU-C 2002 RS, Novate Milanese, Italy) to assess the percentage of dry residue (%RS), evaluated using Formula (1) [43]:

(1) $$\%RS=\frac{\left(c-a\right)}{\left(b-a\right)}\times 100$$

Here,

- = weight of empty tray;
- = weight of the tray with the product before drying;
- = weight of tray with product after drying.

Colours of fresh fruits, vegetable powders, and cookies were evaluated using a digital colourimeter (Minolta, mod. CR-300; Osaka, Japan) according to the CIELab colourimetric system, which identifies the colour of the fruit with three different coordinates: L* (brightness; L* = 0 for black and L* = 100 for white), a* (green/red colour index; a* = −100 for green and a* = +100 for red), and b* (blue/yellow colour index; b* = −100 for blue and b* = +100 for yellow). Values were converted to RGB format using ‘e-paint.co.uk Convert Lab’ software [49]. The colour differences (ΔE) between the fresh fruit and the obtained powder and between the cookies were measured according to Equation (2) [50]:

(2) $$∆E=\sqrt{{\left(L^{*}-L_{0}^{*}\right)}^{2}+{\left(a^{*}-a_{0}^{*}\right)}^{2}+{\left(b^{*}-b_{0}^{*}\right)}^{2}}$$

Here, $L_{0}^{*}$, $a_{0}^{*}$, and $b_{0}^{*}$ represent the values of the colour parameters measured after drying.

The total soluble solids content (TSSC) was measured on the fresh fruit with a digital refractometer (Atago, Tokyo, Japan) and expressed in °Brix while the titratable acidity (TA) was measured with a pH titrator (Titromatic 1S, Crison, Barcelona, Spain) and expressed in grams of citric acid per litre of fruit juice (g citric acid/L^−1^).

#### 2.4.1. Evaluation of Total Phenolic Content (TPC)

Total phenolic content (TPC) was determined using the optimized Folin–Ciocâlteu colourimetric method according to previous research [47,51] with slight modifications. In particular, 0.5 g of each sample of powder was mixed with 10 mL of methanol/water solution (80:20 *v/v*), sonicated for 50 min, and filtered through Whatman 0.45 μm PTFE filters. An aliquot of the filtrate (0.110 mL), 125 μL of a 7% Na_2_CO_3_ solution, and 625 μL of Folin–Ciocâlteu reagent (1:5) were incubated in the dark at 25 °C for 50 min. The Abs was evaluated at 765 nm using a UV-Vis spectrophotometer (Varian Cary 50, Agilent, Santa Clara, CA, USA). Methanol was used as the blank and gallic acid was used for calibration of the standard curve (0.001 to 0.30 mg/mL). TPC was expressed as mg gallic acid equivalents per g (mg GAE g^−1^) of the sample. Data were analysed in triplicate and reported as means ± SEMs.

#### 2.4.2. Radical Scavenging Properties Evaluation, DPPH and ABTS Assay 

The measurement of powder sample antiradical activity follows procedures previously described [52,53]. DPPH and ABTS assays allow us to determine the antioxidant power through the reaction of the sample with a solution of DPPH [2,2-diphenyl-1-picrylhydrazyl] and ABTS [2,2′-azino-bis (3-ethylbenzothiazolino-6-Sulphonic acid] that causes a discolouration of the solution proportional to the antioxidant charge present in the sample. Two grams of sample were extracted using 10 mL of methanol, sonicated for 60 min, and filtered through Whatman 0.45 μm PTFE filters. The filtrate (120 μL) was mixed with 4 mL of DPPH (60 μm) and incubated in the dark at 25 °C for 30 min. Methanol was used as the blank. Scavenging activity was measured at 515 nm using a UV-Vis spectrophotometer (Varian Cary^®^ 50). A calibration curve using Trolox at increasing concentrations [2.5 µm–25 µm] was constructed. The results were reported as Trolox equivalent antioxidant activity (TEAC) and expressed as mmol Trolox equivalents (TEs)/100 g of sample. Five mL of MeOH was added to 2 g of each sample, sonicated, and filtered through Whatman 0.45 μm PTFE filters. The absorbance was read 10 min after the addition of 4 mL of diluted ABTS^+^ to 120 μL of sample. The decrease in absorbance caused by antioxidants, recorded at 734 nm against ethanol, reflected the ABTS^+^ free radical scavenging capacity. A calibration curve using Trolox in a concentration range of 2.5 µm–25 µm was constructed. Obtained values were reported as Trolox equivalent antioxidant activity (TEAC) and expressed as mmol Trolox equivalents (TEs) per 100 g of sample. Data were analysed in triplicate and reported as means ± SEMs.

#### 2.4.3. Polyphenols Extraction

Phenolic compounds phenolics were extracted and analysed using previously modified methods [54,55]. Five grams of sample were homogenized for 45 min in 20 mL of 80% methanol solution using an ultrasound bath. The samples were centrifuged at 5000× *g* for 15 min and the supernatant was recovered. The pellet was re-extracted four times (repeating the protocol described above) and the supernatant was collected and evaporated using a rotary evaporator under vacuum at 45 °C. The residue was redissolved in 1 mL of methanol. This solution, containing free phenolic compounds, was filtered through a 0.22 μm PTFE syringe filter into glass vials before UHPLC-ESI-HRMS analysis. Data were analysed in triplicate and reported as means ± SEMs.

#### 2.4.4. UHPLC-ESI-HRMS Analysis

All the extracts were characterized by UHPLC-ESI-HRMS as reported by Frisina et al. (2023) [56] with slight modifications. The instrument set up consisted of a Dionex Ultimate 3000 RS (Thermo Scientific—Rodano, MI, Italy), interfaced with a high-resolution Q-Exactive orbitrap mass spectrometer (Thermo Scientific, Rodano, MI, Italy) with electrospray ionisation source, operating in negative mode. Chromatographic separation was performed with an Hypersil Gold C18 column (100 mm × 2.1 mm, 1.9 µm particle size) from Thermo Scientific, maintained at a temperature of 30 °C and a flow rate of 300 µL min^−1^. The chromatographic column was equilibrated in 98% solvent A (ultrapure water containing 0.1% of formic acid) and 2% solvent B (methanol). The concentration of solvent B was linearly increased from 2% to 23% in 6 min, remaining isocratic for 5 min, then linearly increased from 23% to 50% in 7 min and from 50% to 98% in 5 min, remaining isocratic for 6 min, and finally returned to 2% in 6 min, remaining isocratic for 3 min. Volume of injected sample was 5 μL. The total run time, including column wash and equilibration, was 38 min. Heated electrospray ionisation (HESI) was selected in negative polarity, with the following operating conditions: 70,000 resolving power (defined as FWHM at *m/z* 200), IT 100 ms, ACG target = 1 × 10^6^, and scan range (100–900 *m/z*). MS/MS analyses were performed according to the following operating conditions: resolution: 35,000, AGC target = 1 × 10^5^, maximum IT 200 ms, and collision energy (stepped NCE): 20, 30, 40. The quadrupole isolation window was set to 2.0 *m/z*. High purity nitrogen was used as the sheath gas (30 arb units) and auxiliary gas (10 arb units). The instrument was calibrated before each analysis using the calibration solution supplied by Thermo Fisher Scientific. Compounds were characterized according to the corresponding HRMS spectra, accurate masses, characteristic fragmentations, and retention times and quantified using calibration curves of analytical reference standards. Standard solutions were daily prepared in methanol. Xcalibur software (version 4.1) was used for instrument control, data acquisition, and data analysis. Individual concentrations of the considered molecules were derived by the external calibration curves of the respective commercial analytical standards. In particular, the concentrations of protocatechuic acid (*m/z* 153.0183), benzoic acid (*m/z* 121.0284), and caffeic acid (*m/z* 179.0342) were obtained with respect to a calibration curve of caffeic acid (r^2^ = 0.9999) in a range between 0.1 and 5 mg/L. A preset kaempferol standard calibration curve (r^2^ = 0.9978) in the concentration range of 0.1–10 mg/L. was used to determine the content of dihydrokaempferol (*m/z* 287.2229). Ellagic acid (*m/z* 300.9991) and quercetin-3-O-glucoside (*m/z* 463.0884) were quantified using the calibration curve of the corresponding reference standards in ranges of 0.1–10 mg/L and 0.5–10 mg/L, respectively.

### 2.5. Sensory Analysis

**Figure 1:**
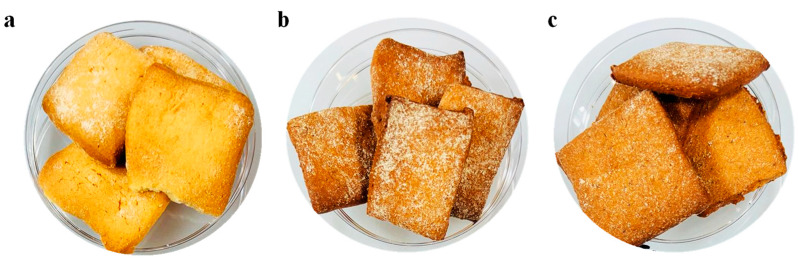
Cookies samples analysed in the sensory panel: (**a**) CTRL-C (control); (**b**) HAD-Cookies (hot air drying); (**c**) US-HAD-Cookies (ultrasound-assisted hot air drying).

The sensory evaluation was conducted by a panel of 14 semi-qualified judges based on 19 descriptors: surface colour (SC), internal colour (IC), colour homogeneity (CO), cookie odour (BO), blackberry odour (MO), burnt odour (BRO), unpleasant odour (UO), hardness (H), softness (S), crunchiness (C), chewiness (CH), cookie taste (BT), blackberry taste (MT), burnt taste (BRT), unpleasant taste (UT), sweetness (SW), bitterness (BI), sourness (SN), and overall acceptability (OA). The evaluation was conducted from 10:00 to 12:00 in a room under white lights. Each panel member received, in random order, a control sample (CTRL-C) and the two samples with the added powders (HAD-Cookies and US-HAD-Cookies) (Figure 1). Between each sample, water was provided for mouth rinsing. The judges rated the intensity of each descriptor on a hedonic scale, assigning score from 1 to 9, representing different levels of intensity of the quality descriptors: 1—no sensation, 2—barely recognisable, 3—very weak, 4—weak, 5—slight, 6—moderate, 7—intense, 8—very intense, and 9—extremely intense.

### 2.6. Statistical Analysis

Results are expressed as means ± SEMs of *n* separate experiments conducted in triplicates. Statistical comparisons were performed by one-way analysis of variance (ANOVA) followed by Tukey’s correction for multiple comparisons. The data were statistically processed using XLStat software version 16.0 for Microsoft Excel (Addinsoft, New York, NY, USA). In all cases, significance was accepted if the null hypothesis was rejected at the *p* < 0.05 level.

## 3. Results

### 3.1. Plant Material

Before the drying process, a physicochemical characterisation was conducted on a sample of 50 fresh fruits (Table 1). A TSSC of 8.36 ± 0.08 °Brix suggests that blackberries have a satisfactory level of soluble sugars, ensuring adequate sweetness. Titratable acidity measures the content of organic acids in fruit, contributing to flavour and shelf life. A TA of 0.29 ± 0.07 g/L indicates that blackberries have a good balance between sweetness and acidity, ensuring a well-rounded flavour profile that enhances overall sweetness. Values of L*, a*, and b* and RGB collectively indicate that blackberries have a dark colour, typical of ripe, high-quality fruit.

### 3.2. Drying Process

Table 2 presents the results of drying experiments conducted on blackberries using different treatment methods at 75 °C. The values represent the percentages of dry residue (%RS) obtained after specified durations, illustrating the efficiency of each method in preserving fruit integrity.

The results show that HAD treatment at 75 °C produced a dry residue (%RS) of 11.72 ± 0.74 after 7 h while the US-HAD treatment, at the same temperature, resulted in a dry residue (%RS) of 10.37 ± 1.67 after 6 h. 

### 3.3. Chemical-Physical Analysis

An important quality parameter for consumers is the visual appearance of the fruit. Table 3 illustrates the colour differences (ΔE) in blackberry powders obtained from both treatments compared to fresh blackberries.

**Figure 2:**
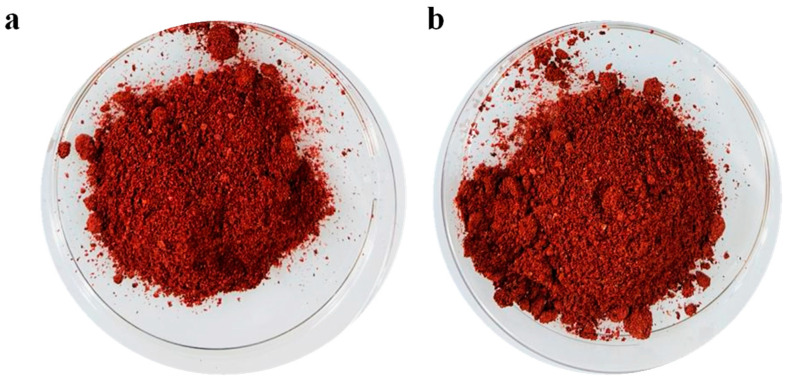
Visual characteristics of the blackberry vegetable powders analysed: (**a**) blackberry powder HAD-BP (hot air drying); (**b**) blackberry powder US-HAD-BP (ultrasound-assisted hot air drying).

The results show that the blackberry powder samples obtained through the two drying treatments exhibited significant colour differences compared to fresh blackberries, as indicated by the ΔE values. The powder from hot air drying (HAD-BP) had a ΔE value of 28.27 ± 0.78, indicating a substantial colour difference from the fresh blackberries. Conversely, the powder from ultrasound-assisted hot air drying (US-HAD-BP) had a ΔE value of 26.70 ± 0.44. Although this also signified a significant colour difference from the fresh blackberries, it was less pronounced than that observed in the HAD-BP sample. It was also visually evident that US-HAD-BP caused significantly less colour variation than HAD-BP, as shown in Figure 2.

**Figure 3:**
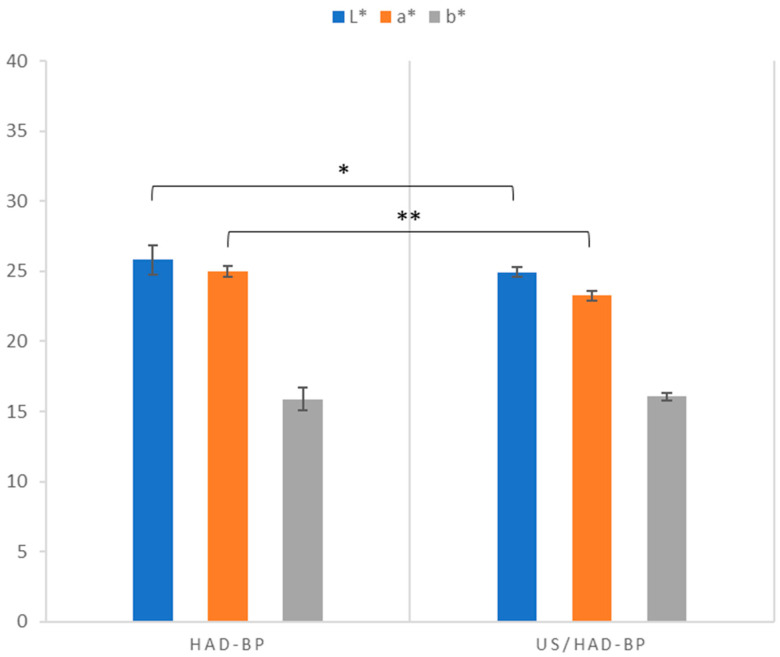
CIELab* values of blackberry powders (HAD-BP and US-HAD-BP) after drying and mill. Results indicate the mean values ± standard deviations. Asterisks indicate statistically significant differences between treatments with * for *p* ≤ 0.05, ** for *p* ≤ 0.01; no asterisk indicates a non-significant difference.

Figure 3 shows the values of the colourimetric parameters L* (brightness), a* (red/green), and b* (yellow/blue) for blackberry powders obtained by two drying methods (HAD and US-HAD). The L* values for the HAD-BP and US-HAD-BP powders were 25.80 ± 1.04 and 24.94 ± 0.36, respectively, indicating a significantly lower brightness, with reference to Table 1, but closer to the values of the fresh samples for US-HAD treatment. The parameter a* showed a more marked difference compared to the fresh samples (Table 1), with values of 24.99 ± 0.38 for HAD-BP and 23.25 ± 0.35 for US-HAD-BP. The statistical analysis performed also showed significant differences between the two treatments. For the parameter b*, the values were similar: 15.87 ± 0.82 for HAD-BP and 16.05 ± 0.26 for US-HAD-BP, but significantly different from the fresh samples (1.94).

Table 4 displays the colour difference (∆E) values observed in cookies containing blackberry powder, comparing two different drying treatments. The ∆E values quantify the perceptible differences in colour between the control cookies and those processed with the HAD and US-HAD methods, providing insights into the impact of drying techniques on final product appearance.

Table presents the colour difference values (∆E) for three samples of cookies. The HAD-Cookies sample exhibited a colour difference of 22.55 ± 1.19 compared to the control cookies whereas the US-HAD-Cookies sample showed a larger difference of 31.06 ± 1.32. 

**Figure 4:**
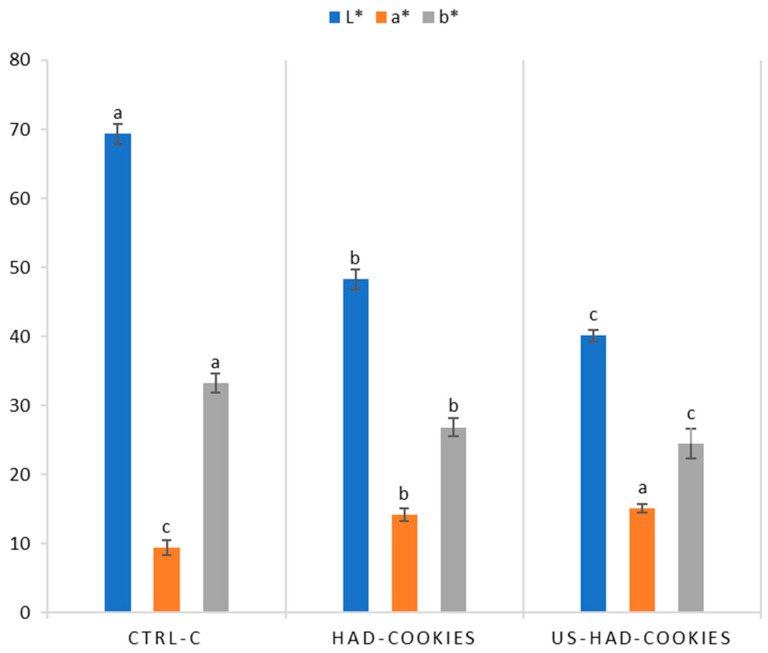
CIELab* values of blackberry cookies (HAD-Cookies and US-HAD-Cookies) after baking. Results indicate the mean values ± standard deviations. Values in the histograms followed by a different letters (a, b, c) are significantly different (*p* < 0.05).

In addition, Figure 4 illustrates the colourimetric parameters L*, a*, and b* of cookies containing 10% blackberry powder. Both treatments showed significant differences in CIELab values compared to the control (CTRL-C). The US-HAD-Cookies sample exhibited a lower L* value (40.12 ± 0.86) than the HAD-Cookies sample (48.23 ± 1.47) when compared to CTRL-C (69.30 ± 1.40). Specifically, a 30% loss was observed for HAD-Cookies and a 42% loss for US-HAD-Cookies, highlighting a significant difference between the two treatments. The a* parameter was higher in US-HAD-Cookies (15.13 ± 0.61) than in HAD-Cookies (14.14 ± 0.92), but both were lower than CTRL-C, with respective losses of 25% and 30%. Similarly, the b* parameter was higher in HAD-Cookies (26.85 ± 1.26) than in US-HAD cookies (24.46 ± 2.22), with a significant difference between treatments and compared to the control. In fact, both HAD-Cookies and US-HAD-Cookies showed reductions of 32% and 38%, respectively, compared to CTRL-C.

### 3.4. Evaluation of Total Phenolic Content (TPC) and Radical Scavenging Properties 

The total phenolic content (TPC) and radical scavenging activity values of HAD-BP, US-HAD-BP, and “00” control flour (CTRL-P) and of their 10% fortified cookies (HAD-Cookies, US-HAD-Cookies) and control cookies (CTRL-C) were measured. The hot-air-dried samples (HAD-BP) showed lower values of both antiradical activity and total polyphenolic content compared to the hot-air-dried flour sample combined with ultrasound (US-HAD-BP). 

In particular, as shown in Table 5, a higher TPC value was highlighted in the US-HAD-BP sample (33.054 mgGAE/g) compared to the HAD-BP (32.111 mgGAE/g) and “00” flour samples (CTRL) (3.676 mgGAE/g). The highest increase in antiradical activity was also observed in US-HAD-BP with values of 49.067 mmol TE/100 g and 42.632 mmol TE/100 g for the DPPH and ABTS tests, respectively, while the lowest was recorded for the control flour (CTRL-P) (3.915 and 4.423 mmol TE/100 g for the DPPH and ABTS tests, respectively). 

Statistical analysis showed significantly increases in all experimental treatments compared to the controls, especially for the US-HAD-BP sample, both for antioxidant activity and antiradical activity. The HAD-BP and US-HAD-BP samples did not show significant differences from each other for all tests performed.

Same analyses were conducted on cookies fortified with 10% HAD-Cookies and on cookies fortified with 10% US-HAD-Cookies. The addition of the blackberry powder to the “00” flour significantly improved the antiradical and antioxidant activity of the samples. As shown in Table 6, the fortified US-HAD-Cookies had higher values of TPC (13.882 mgGAE/100 g) and antiradical activity (9.232 and 8.536 mmol TE/100 g for the DPPH and ABTS tests, respectively) compared to the control cookie (CTR-C) products with only “00” flour (2.326 and 3.101 mmol TE/100 g for the DPPH and ABTS tests, respectively).

Analysis showed significant increases in all experimental treatments compared to controls, especially for the US-HAD-Cookies sample, both for antioxidant activity and antiradical activity. The HAD-Cookies and US-HAD-Cookies samples did not show significant differences from each other for all tests performed.

### 3.5. Evaluation of Phenolic Compounds

UHPLC-ESI-MS analysis of the polyphenolic profile of the fresh blackberry fruits (F. Blackberries) showed (Table 7) an abundance of phenolic acids, organic acids, and flavonoids; most detected compounds were protocatechuic acid (123.3 mg/100 g), ellagic acid (292.25 mg/100 g), quercetin 3-O-glucoside (678.1 mg/100 g), and dihydrokaempferol (276.4 mg/100 g). The two blackberry powders prepared with two different HAD (HAD-BP) and US + HAD (US-HAD-BP) treatments showed lower values compared to the fresh fruit but a qualitatively higher profile than the control flour of type “00” (CTRL-P). Samples subjected to the US-HAD treatment showed slightly higher values, especially for ellagic acid (78.67–38.9 mg/100 g for US-HAD-BP and HAD-BP). The quali-quantitative analysis of fortified cookies (HAD-Cookies and US-HAD Cookies) highlighted a slight increase compared to the control cookies. In this case, the values of the phenolic compounds detected were similar to each other, except for benzoic acid (0.9–1.8 mg/100 g for HAD-Cookies and US-HAD-Cookies, respectively). Protocatechuic acid and caffeic acid, absent in the control cookies (CTRL-C), were instead revealed in the HAD-Cookies and US-HAD-Cookies samples, assuming that their presence was only due from the fortification with blackberry powder.

### 3.6. Sensory Analysis

**Figure 5:**
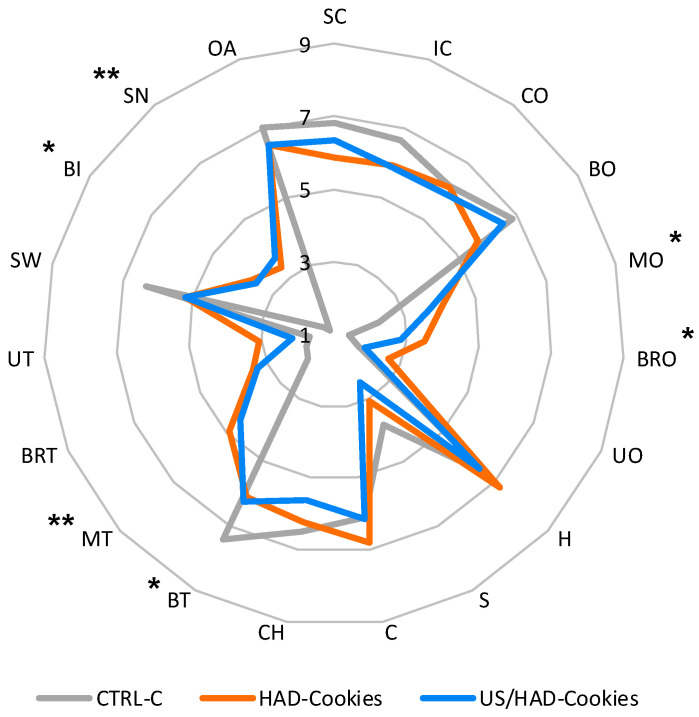
Sensory evaluation of blackberry cookies (HAD-Cookies and US-HAD-Cookies) after baking. The spider plot indicates the average intensity values of the descriptors, ascribed by judges, on a hedonic scale from 1 to 9. Asterisks indicate statistically significant differences between treatments with * for *p* ≤ 0.05, ** for *p* ≤ 0.01; no asterisk indicates a non-significant difference. Legend: SC (surface colour); IC (internal colour); CO (colour homogeneity); BO (cookie odour); MO (blackberry odour); BRO (burnt odour); UO (unpleasant odour); H (hardness); S (softness); C (crispness); CH (chewiness); BT (cookie taste); MT (blackberry taste); BRT (burnt taste); UT (unpleasant taste); SW (sweetness); BI (bitterness); SN (acidity); OA (overall acceptability).

The sensory evaluation of the cookies was conducted 24 h after baking. The results are shown in Figure 5. From the results obtained, it can be observed that about the visual descriptors (SC, IC, CO), the judges, although there was a clear difference in colouring between the control (CTRL-C) and theses (HAD and US-HAD), evaluated these descriptors positively in the treated samples; in fact, no significant difference was found compared to the control. In the olfactory descriptors, the cookie odour (BO) was found in all samples while the blackberry odour (MO) was only detected in the HAD thesis. For the taste descriptors, important results can be seen in the texture. In particular, the descriptors H, S, C, and CH showed no differences between the samples, indicating an appreciation, but above all that, the addition of the powders did not cause a change in the rheological and mechanical characteristics of the treated samples compared to the CTRL-C sample. This is of critical importance, both for shelf life but also because it makes it possible to standardise production and place on the market products that, in terms of texture, are perfectly identical to a classic cookie made with 100% wheat flour ‘‘00’‘. However, significant differences were found in the blackberry flavour (BT), indicating that the addition of 10% of the powder was sufficient for the characteristic taste of the small fruit to be detected by the judges. Confirming this, the SN descriptor was reported to be intense in line with the classic taste of fresh blackberry fruits. Negative descriptors such as UO and UT received the lowest scores, so, in addition to the health aspects, we can confirm that the addition of the powders was also highly appreciated from a sensory point of view with no significant difference between the HAD-Cookies and US-HAD-Cookies samples.

## 4. Discussions

TSSC is a critical indicator of fruit sweetness. The value of TSSC is crucial for assessing fruit quality for both fresh consumption and processing as a higher TSSC enhances flavour and perceived sweetness in the final product [57]. Our data showing a TSSC of 8.36 ± 0.08 °Brix confirmed that the analysed blackberries had a substantial level of sweetness, making them suitable for both direct consumption and processing. With a TA of 0.29 ± 0.07 g/L, our findings indicate that the blackberries had a good balance of sweetness and acidity, which not only contributed to their flavour but also helped in maintaining microbial stability [14,58]. The values of L*, a*, b*, and RGB collectively indicated that the blackberries possessed a dark colour, typical of ripe fruits (Table 1) [14]. This colouration is significant not only for visual appeal but also for antioxidant and nutritional properties as a darker colour is often associated with higher contents of anthocyanins and other beneficial phenolic compounds [59]. The data confirmed that the analysed blackberry fruits were suitable for both direct consumption and processing, affirming their quality for intended uses. These findings are consistent with those of Hassimotto et al. (2008) and Schulz et al. (2019) [60,61], highlighting their ideal maturity and quality for consumption and processing.

Drying is a critical process in food preservation, with the aim of removing moisture from the fruit while maintaining its nutritional content and sensory attributes. Effective methods of drying play a crucial role in maintaining product quality, including texture, flavour, and colour [62]. Moreover, shorter drying durations minimize prolonged exposure to high temperatures, which can degrade bioactive compounds such as vitamins, anthocyanins, and phenols [63]. This preservation helps maintain the nutritional value, colour, flavour, and texture of the food product. Our results indicate that pre-treatment with ultrasound reduced the drying time by one hour to achieve a comparable dry residue. Thus, ultrasound-assisted hot air drying (US-HAD) technology emerges as a preferred option for the food industry, combining operational efficiency and sustainability while optimally preserving product characteristics.

An important quality parameter for consumers is the visual appearance of the fruit. Colour is the attribute that most influences consumers when purchasing fruits [64]. In fact, it is considered the most important visual attribute in quality perception [65]. Fruit drying, as reported in the literature [26,66,67], influences changes in the texture, flavour, and taste and also in the characteristic colour of the fruits subjected to the process. Table 3 illustrates the colour differences (ΔE) in blackberry powders obtained from both treatments compared to fresh blackberries. The drying process induced a colour difference (ΔE) signifying an alteration of the overall colour in both treatments. These results indicate that ultrasound treatment (US-HAD-BP) causes significantly less colour variation compared to conventional treatment (HAD-BP) (Figure 2). This significant difference in the a* value of US-HAD-BP powder suggests that ultrasound pre-treatment helped preserve the red component better in the final powders due to the presence of anthocyanins. Pre-treatment with ultrasound improves the preservation of the red component in US-HAD-BP powder as it facilitates a faster extraction of water from fruits, thus minimising their exposure to heat and light, factors that contribute to their degradation [68]. This process also helps stabilise the anthocyanins, ensuring their subsequent processing at lower temperatures during drying, thus preserving the integrity of these heat-sensitive compounds. Reduced colour variation is generally preferred as it suggests the better preservation of the original colour, which is a key indicator of visual quality and may reflect the lesser degradation of pigmented compounds such as anthocyanins [69,70]. Anthocyanins are sensitive to various physical and chemical treatments including high temperatures [70]. Ultrasound, however, may have helped preserve these compounds better than conventional HAD treatment, as well as reducing the general browning caused by the drying process due to the shorter treatment time. This hypothesis is supported by previous studies showing that ultrasound can increase cell permeability, improving the efficiency of the drying process and preserving bioactive compounds [71]. Li et al. (2020) [72] reported that ultrasound treatment with different powers for 20 min significantly improved the values of L*, a*, and b* in wine samples by stabilising the colour loss. This indicates that the yellow component of the powders remained unchanged between the two treatments, suggesting that ultrasound had no significant effect.

Also, baking is a complex process that induces physical, chemical, and biochemical changes in the grain matrix, including volume expansion, water evaporation, the formation of a porous structure, protein denaturation, starch gelatinisation, crust formation, and browning [73]. The colour of the cookie surface is a fundamental characteristic closely linked to aroma, texture, and appearance—critical aspects for consumers. These findings indicate that the HAD-Cookies sample closely resembled the colour of the control cookies, experiencing less colour variation compared to the US-HAD-Cookies sample. The interaction of anthocyanins with other ingredients in the cookie matrix, such as proteins and fats, can influence the final colour [74]. The better-preserved anthocyanins from the ultrasound pre-treatment might interact differently during baking, causing a higher colour difference. In fact, the better preservation of the a* parameter after baking in the HAD-US-Cookies samples could be attributed to the higher preservation of anthocyanins. Considering that the control sample represented the standard consumer reference colour, it is likely that consumers would prefer the HAD-Cookies sample over the US-HAD-Cookies sample due to their closer resemblance in colour to the standard. Often, colour serves as an indicator of baking degree. The formation of colour and aroma in the crust of bakery products during baking primarily results from the Maillard reaction [75]. These data demonstrate the consistent trend in results across all colourimetric parameters influenced by the baking process, albeit with higher values due to baking. 

Our results revealed also that the incorporation of blackberry powder into cookie formulas led to significant increases in antioxidant activity, total phenolic content (TPC), and polyphenolic profile proportionate to the percentage replacement of blackberry powder used in the production recipe. Results obtained from the evaluation of total phenolic content (TPC) and radical scavenging activity were in accordance with those obtained by Gil-Martínez et al. (2023) [76], which highlighted TPC values for blackberries equal to 31.1 ± 4.9 mgGAE/g and antiradical activity values equal to 57.6 ± 8.3 mmol TEAC/100 g; in this study, the blackberries were dried at 45 °C and the ground dry material was extracted with an ethanol/water solution (50:50 *v/v*) at 50 °C with constant stirring for 14.5 h. Albert et al. (2022) [77] found a similar range of antioxidant activity values (21.43–33.11 mg GAE/g) on fresh frozen blackberries extracted with different solvents (80% ethanol, 70% acetone + 2% acetic acid, 60% methanol + 3% formic acid, 90% acetonitrile + 10% HCl 6 M). Similar values were found in the work of Jazic’ et al. (2018) [78]; the authors researched antioxidant activity in the ethanolic extracts of previously dried wild and cultivated blackberries. The results showed a range of close values between 21.59 and 40.18 mg GAE/g. Dai et al. (2007) [79], however, obtained lower levels of TPC (17.32 mgGAE/g) and antiradical activity (66.98 μmol TEAC/g d.w.). In this study, frozen blackberry puree was freeze-dried and extracted in ethanol acidified with 0.01% HCl. Santos et al. (2023) [80], for example, analysed an extract using ultrasound-assisted extraction, reporting slightly higher values equal to 52.36 mg GAE/g for TPC and for antiradical activity (55.56–60.86 mmol TEAC/100 g for DPPH and ABTS tests, respectively). Another study evaluated TPC in blackberries using near-infrared spectroscopy (NIRS) and showed values between 17.36 and 36.78 mgGAE/g [81]. 

In a recent study by Sik et al. (2024) [82], the antioxidant properties of wild blackberry and its possible use as a functional ingredient for the production of fortified muffins were evaluated. The work involved ultrasound-assisted methanolic extraction acidified with 0.5% HCl. The total polyphenol content of the fruits was equal to 53.8 mg GAE/g; this process highlighted an increase in antioxidant activity and greater acceptability in the finished product. The results of the study suggest that blackberries can be used as functional ingredients to increase the antioxidant activity values of a food [82]. Higher phenolic acid values than those found in the literature were highlighted in fresh blackberry fruits. In particular, Sellappan et al. (2002) [83] found ellagic acid values in a range between 30.01 and 30.8 mg/100, similar to those found (35.7–54.7 mg/100 g) by DJurić et al. (2014) [84]. In the study by Da Silva et al. (2018) [85], the beneficial health effects attributable to the polyphenols present in blackberries were evaluated and the availability of phenolic compounds after the transformation of blackberries into jam was investigated. Significantly fewer phenolic compounds were identified in the jam, indicating degradation during the heating process; this could also explain the significant loss of phenolic compounds in the samples analysed in this study. However, the finished product improved its antioxidant activity (ABTS assay) of the processed products after storage respect to the control, which which may be related related to the development of new compounds with higher antioxidant activity. Therefore, the transformation of blackberries into food products and fortified foods represents a valid alternative to increase the antioxidant activity of the finished product [85].

Finally, the sensory evaluation shows that the addition of blackberry powder was well received by the judges, with positive ratings for visual, aromatic, and taste attributes. The negligible differences between the HAD-Cookies and US-HAD-Cookies samples further emphasise the effectiveness of ultrasound pre-treatment in maintaining sensory quality attributes comparable to conventional drying methods. Belščak-Cvitanović–Zoran and Opalìc (2013) [86] demonstrated that pear samples subjected to longer ultrasound treatments (35 and 45 min) and dried for extended periods show darker colouring, harder texture, and the presence of cracks, leading to lower sensory acceptability. This effect could probably also be transferred to foods when these products are used as ingredients as in our case. These results not only validate the sensory appeal of blackberry-enriched cookies but also suggest potential for the standardised production and marketability of fruit-enriched baked goods.

## 5. Conclusions

In this study, the effects of the addition of blackberry powder dried by two different methods, hot air drying (HAD) and ultrasound-assisted hot air drying (US-HAD), on the physical and sensory characteristics of functional cookies were examined. The US-HAD method showed a significant reduction in drying time compared to the conventional HAD method, saving approximately one hour of time and energy to reach a final moisture content of about 12%. The results showed that cookies enriched with blackberry powders, both HAD and US-HAD, presented a significant increase in antioxidant activity compared to the control cookies (CTRL-C). The values of antioxidant activity, measured with the ABTS and DPPH tests, were higher than those of the control cookies. UHPLC-ESI-MS analysis revealed an increase in polyphenol content in the fortified cookies, with the main phenolic compounds detected including protocatechuic acid and dihydrokaempferol. Fortified cookies showed promising values of total phenolic content (TPC), directly associated with improved antioxidant properties. The sensory evaluation showed that cookies fortified with US-HAD powder scored higher in terms of blackberry flavour and general acceptability than HAD and control cookies. This suggests that the use of US-HAD technology not only improves the nutritional but also the sensory properties of the cookies. The incorporation of dried blackberry powders, especially those obtained by the US-HAD method, into functional cookies not only reduces food waste but also enriches the nutritional profile of baked goods, offering a healthy and sustainable alternative in the use of damaged fruits. The absence of added sugar and fat in blackberry powder has significant health benefits for consumers, reducing the intake of potentially unhealthy ingredients and promoting a more balanced diet. From an economic point of view, the food industry can capitalise on the growing demand for healthy, fortified foods by positioning products enriched with dried blackberry powder as attractive and nutritionally advantageous options for health-conscious consumers.

## Figures and Tables

**Table 1:** Quality characteristics of fresh blackberry fruits: TSSC (total soluble solids content, °Brix); TA (titratable acidity, g/L^−1^ citric acid); L* (brightness); a* (red); b* (yellow); RGB (conversion of CIELab* values). Values are represented as means ± standard deviations of the data obtained.

	TSSC (°Brix)	TA (g Citric a./L^−1^)	L*	a*	b*	RGB
F. Blackberries	8.36 ± 0.08	0.29 ± 0.07	18.72 ± 0.74	1.46 ± 0.27	1.95 ± 0.72	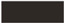

**Table 2:** Results in terms of drying time and dry residue of blackberries. °C represents the treatment temperature, *h* represents the treatment time expressed in hours, and %RS represents the percentage of dry residue. The values of %RS are expressed as the means ± standard deviations of the results obtained. Different letters represent statistically significant differences (*p* ≤ 0.05).

Samples	h	°C	%RS
HAD	7	75	11.72 ± 0.74 ^a^
US-HAD	6	75	10.37 ± 1.67 ^b^

**Table 3:** Colour differences (Δ*E*) of blackberry powders measured on the fresh samples after drying process. RGB column represents the conversion of CIELab* values. Values are represented as means ± standard deviations of the data obtained. Different letters represent statistically significant differences (*p* ≤ 0.05).

Samples	∆*E*	RGB
F. Blackberries		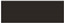
HAD-BP	28.27 ± 0.78 ^a^	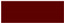
US-HAD-BP	26.70 ± 0.44 ^b^	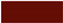

**Table 4:** Colour difference (Δ*E*) values of blackberry cookies measured on the samples after baking. RGB column represents the conversion of CIELab* values. Values are represented as means ± standard deviations of the data obtained. Different letters represent statistically significant differences (*p* ≤ 0.05).

Samples	∆*E*	RGB
CTRL-C		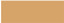
HAD-Cookies	22.55 ± 1.19 ^a^	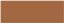
US-HAD-Cookies	31.06 ± 1.32 ^b^	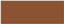

**Table 5:** Antioxidant and antiradical activity of blackberry powders prepared with two different treatments, HAD (HAD-BP) and US + HAD (US-HAD-BP), and “00”-type control flour (CTRL-P). Results indicate mean values ± SEMs. Data within a column followed by different letters were significantly different according to Tukey’s test.

	CTRL-P	HAD-BP	US-HAD-BP	SEM	*p* Value
TPC mgGAE/g	2.972 ^a^	32.111 ^b^	33.054 ^b^	4.948	<0.0001
DPPH mmolTEAC/100 g	3.915 ^a^	48.484 ^b^	49.067 ^c^	7.477	<0.0001
ABTS mmolTEAC/100 g	4.423 ^a^	39.576 ^b^	42.632 ^c^	6.130	<0.0001

**Table 6:** Antioxidant and antiradical activity of fortified cookies prepared with two blackberry powders—HAD-Cookies and US-HAD-Cookies—and “00”-type control cookies (CTRL-C). Results indicate mean values ± SEMs. Data within a column followed by different letters were significantly different according to Tukey’s test.

	CTRL-C	HAD-Cookies	US-HAD-Cookies	SEM	*p* Value
TPC mgGAE/g	2.232 ^a^	13.033 ^b^	13.882 ^c^	1.753	<0.0001
DPPH mmolTEAC/100 g	2.326 ^a^	8.792 ^b^	9.232 ^c^	1.116	<0.0001
ABTS mmolTEAC/100 g	3.101 ^a^	8.049 ^b^	8.536 ^c^	0.653	<0.0001

**Table 7:** Phenolic compound evaluation of fresh blackberry fruits and blackberry powders prepared with two different treatments—HAD (HAD-BP) and US + HAD (US-HAD-BP)—and “00”-type control flour (CTRL-P) and of fortified cookies prepared with two blackberry powders—HAD-Cookies and US-HAD-Cookies—and “00”-type control cookies (CTRL-C). Results indicate mean values ± SEMs; n.d.: not detected.

	F. Blackberries	CTRL-P	HAD-BP	US-HAD-BP	CTRL-C	HAD-Cookies	US-HAD-Cookies
	mg/100 g						
*Phenolic acids*							
Protocatechuic acid	123.3	n.d.	34.6	34.3	n.d.	3.1	3.2
*p*-coumaric acid	37.3	n.d.	n.d.	n.d.	n.d.	n.d.	n.d.
Ellagic acid	292.25	n.d.	38.9	78.67	n.d.	n.d.	n.d.
*Organic acids*							
Benzoic acid	20.1	n.d.	0.4	1.2	0.5	0.9	1.8
Caffeic acid	92.55	n.d.	n.d.	3.22	n.d.	n.d.	3.22
*Flavonoids*							
Quercetin-3-O-glucoside	678.1	n.d.	n.d.	n.d.	n.d.	n.d.	n.d.
Dihydrokaempferol	276.4	18.6	38.2	25.4	17.6	21.2	20.9

## Data Availability

The original contributions presented in the study are included in the article, further inquiries can be directed to the corresponding author.
